# An Epstein-Barr Virus-Encoded Protein Complex Requires an Origin of Lytic Replication *In Cis* to Mediate Late Gene Transcription

**DOI:** 10.1371/journal.ppat.1005718

**Published:** 2016-06-27

**Authors:** Reza Djavadian, Ya-Fang Chiu, Eric Johannsen

**Affiliations:** 1 Department of Medicine, University of Wisconsin School of Medicine and Public Health, Madison, Wisconsin, United States of America; 2 Department of Oncology (McArdle Laboratory for Cancer Research), University of Wisconsin School of Medicine and Public Health, Madison, Wisconsin, United States of America; 3 Research Center for Emerging Viral Infections, Chang Gung University, Taoyuan, Taiwan; 4 Department of Microbiology and Immunology, Chang Gung University, Taoyuan, Taiwan; 5 Department of Medical Laboratory, Chang Gung Memorial Hospital, Linkou, Taiwan; Wistar Institute, UNITED STATES

## Abstract

Epstein-Barr virus lytic replication is accomplished by an intricate cascade of gene expression that integrates viral DNA replication and structural protein synthesis. Most genes encoding structural proteins exhibit “true” late kinetics–their expression is strictly dependent on lytic DNA replication. Recently, the EBV *BcRF1* gene was reported to encode a TATA box binding protein homolog, which preferentially recognizes the TAT**T** sequence found in true late gene promoters. *BcRF1* is one of seven EBV genes with homologs found in other β- and γ-, but not in α-herpesviruses. Using EBV BACmids, we systematically disrupted each of these “βγ” genes. We found that six of them, including *BcRF1*, exhibited an identical phenotype: intact viral DNA replication with loss of late gene expression. The proteins encoded by these six genes have been found by other investigators to form a viral protein complex that is essential for activation of TATT-containing reporters in EBV-negative 293 cells. Unexpectedly, in EBV infected 293 cells, we found that TATT reporter activation was weak and non-specific unless an EBV origin of lytic replication (*Ori*Lyt) was present *in cis*. Using two different replication-defective EBV genomes, we demonstrated that *Ori*Lyt-mediated DNA replication is required *in cis* for TATT reporter activation and for late gene expression from the EBV genome. We further demonstrate by fluorescence in situ hybridization that the late BcLF1 mRNA localizes to EBV DNA replication factories. These findings support a model in which EBV true late genes are only transcribed from newly replicated viral genomes.

## Author Summary

A strict linkage between viral DNA replication and the onset of late gene expression is commonly found in DNA viruses. In herpesviruses, the precise mechanism for this requirement remains elusive. Competing models are titration of putative repressors as a result of increase in template copy number and the requirement of newly replicated DNA as the template for late gene transcription. Efforts to differentiate between these two models have been hampered in part by the use of reporter assays which may not recapitulate the effects of whole viral genomes. Several reports, using reporter assays, provide conflicting evidence arguing for both models in EBV. By performing experiments in the context of the whole virus, we demonstrate that EBV late gene expression requires both the products of six genes unique to β- and γ-herpesviruses and the presence of EBV origin of lytic replication (*Ori*Lyt) *in cis*. These findings provide strong evidence that the second model is correct for EBV, i.e., the newly replicated viral genome serves as the template for EBV late gene transcription.

## Introduction

Epstein-Barr virus (EBV) is a γ-herpesvirus that infects more than 95% of the human adult population. Primary infection is usually asymptomatic if acquired early in life, but often results in infectious mononucleosis in adolescence [1,2]. EBV is associated with several B-cell and epithelial cancers, including Burkitt lymphoma, Hodgkin lymphoma, post-transplant lymphoproliferative disorder, nasopharyngeal and gastric carcinoma [3–6]. Like all herpesviruses, EBV exists in two states in infected cells: latent infection and lytic replication. In latent infection, only a limited subset of viral genes is expressed, and infectious virions are not produced. In contrast, during lytic replication (or productive infection), nearly all viral genes are transcribed, viral DNA is replicated, and infectious virions are produced, enabling transmission to other cells and hosts. Although EBV-positive tumors are characterized by latent infection, there is increasingly compelling evidence that both latent infection and lytic gene expression are essential for emergence of EBV-associated malignancies [7–10].

As with all herpesviruses, EBV lytic replication proceeds through an ordered cascade of gene expression. First, immediate-early genes *BRLF1* and *BZLF1*, encoding the transcription factors R (also called Rta) and Z (also called Zta, ZEBRA or EB1), respectively, are expressed [11–16]. R and Z activate the promoters of early genes [12,17–19]. Many early genes encode proteins directly or indirectly involved in viral DNA replication, which is initiated at two origins of lytic replication (*Or*iLyt) used during the productive cycle of EBV [20]. Following viral DNA synthesis, late genes are expressed. EBV late genes mainly encode structural proteins of the EBV virion that elicit strong immune responses (reviewed in reference [11]). EBV late genes can be further subdivided into two groups based on the degree of dependence on viral DNA replication. True late genes absolutely require viral DNA replication for their expression, while leaky late (or delayed early) genes, although usually expressed after DNA replication, can still be expressed from viruses defective for DNA replication. True late genes are often distinguished by their inability to be expressed in presence of viral DNA polymerase inhibitors such as acyclovir [21]. While regulation of immediate-early and early gene expression in herpesviruses is well characterized, relatively little is known about regulation of late genes.

In contrast to all other viral gene promoters, the promoters of EBV true late genes (simply referred to as late genes hereafter) frequently contain a non-canonical TATA box with a thymidine at the fourth position (TAT**T**) [22]. This non-canonical TATT sequence has been recently identified as a critical element for activation of late genes in the β-herpesvirus human cytomegalovirus (HCMV), as well as other γ-herpesviruses, including Kaposi’s sarcoma-associated herpesvirus (KSHV) and murine herpesvirus-68 (MHV-68) [23–25]. An important advance in understanding of EBV late gene regulation came with the prediction that EBV *BcRF1* encodes a TATA box binding protein (TBP)-homolog that preferentially recognizes the TATT box-containing promoters [26]. This prediction was subsequently confirmed experimentally; furthermore, deletion of *BcRF1* from the EBV genome was found to inhibit late gene expression without impairing lytic DNA replication [27]. *BcRF1* is one of 7 genes (*BcRF1*, *BDLF3*.*5*, *BDLF4*, *BFRF2*, *BGLF3*, *BTRF1*, and *BVLF1)* with homologs found in β- and γ- but not in α-herpesviruses (referred to as βγ genes hereafter). Interestingly, in both MHV-68 and HCMV, 5 out of 7 βγ genes, including the *BcRF1* homologs, have been reported to produce the identical phenotype when deleted: 1) intact viral DNA synthesis; and 2) impaired late gene expression [28–35]. In EBV, two additional βγ gene knockout viruses deleted for *BDLF4* and *BFRF2* have been characterized and found to exhibit this specific defect in late gene expression [36,37]. Additionally, in reporter assays, 6 EBV βγ genes (all but *BTRF1*) were required together to activate a TATT-driven luciferase construct in EBV-negative 293 cells [37]. The requirement for *BDLF3*.*5*, *BGLF3*, *BTRF1*, and *BVLF1* for late gene expression in the context of the viral genome is unknown.

The existence of a strict link between late gene transcription and the onset of viral DNA replication is observed among many DNA viruses. In herpesviruses, a critical unresolved question in understanding this link is whether an origin of lytic replication is required *in cis* with the late gene or if DNA replication *in trans* is sufficient to permit late gene expression. For example, in SV40, DNA replication *in trans* is sufficient to titrate transcriptional repressors that block late gene expression from the SV40 genome at early stages of replication [38]. In contrast, a requirement for an *Ori*Lyt *in cis* implies the parental genome is not competent for late gene transcription and that newly replicated DNA serves as the template for production of late gene mRNAs.

Attempts to resolve whether *Ori*Lyt is required *in cis* or *in trans* have met with conflicting results, in part because studies using plasmids do not always recapitulate effects in the context of whole viral genomes [39]. In the case of MHV-68, it was shown that a reporter plasmid containing a late promoter could not be activated unless *Ori*Lyt was present on the plasmid [25]. Studies in KSHV suggest that activation of KSHV K8.1 late promoter can be enhanced by the left-end viral origin of replication, *Ori*Lyt-L, largely *in trans* [24]. The requirement of *Ori*Lyt-mediated DNA replication *in cis* or *in trans* for late gene expression is also controversial in the EBV field. Using a transient late promoter-reporter system, Serio *et al*. showed that although activity of such reporters required EBV lytic replication occurring in the same cell, *Ori*Lyt-mediated DNA replication was not required *in cis* [22]. In contrast, Amon *et al*. demonstrated that robust late gene expression was detected in EBV-positive cell lines stably expressing late-promoter reporter plasmids only when EBV *Ori*Lyt was present on the reporters [40]. Finally, studies by Aubry *et al*. describe a viral pre-initiation complex (vPIC) encoded by 6 βγ genes (*BcRF1*, *BDLF3*.*5*, *BDLF4*, *BFRF2*, *BGLF3*, and *BVLF1*) that activates a TATT-containing promoter reporter plasmid lacking an *Ori*Lyt in EBV negative cells [37]. Thus, the requirement for *Ori*Lyt-mediated DNA replication for EBV late gene expression appears to depend upon the model system employed and has not been examined in the context of the intact EBV genome.

Here, we used EBV BACmids to analyze the role of each βγ gene in late gene expression. Using cell lines stably infected with EBV BACmids individually mutated for each βγ gene, we demonstrate that 6 out of 7 (*BcRF1*, *BDLF3*.*5*, *BDLF4*, *BFRF2*, *BGLF3*, and *BVLF1*) βγ genes are required for late gene expression from the virus, but dispensable for DNA replication. In reporter assays, βγ gene mediated activation of late-gene promoters required thymidine at the forth position of the non-canonical TAT**T** sequence and, importantly, strictly required *Ori*Lyt-mediated DNA replication. Furthermore, using replication-defective EBV BACmids (lacking the single-stranded DNA binding protein BALF2 or *Ori*Lyt), we found that *Ori*Lyt-mediated DNA replication *in cis*, and not *in trans*, is a pre-requisite for EBV late gene expression. Finally, using fluorescence in situ hybridization and a visible EBV derivative, we demonstrate that the BcLF1 late mRNA localizes to the EBV replication factories. Our results are consistent with a model in which the viral pre-initiation complex encoded by 6 EBV βγ genes mediate late gene transcription from newly replicated viral DNA.

## Results

### Six of the seven EBV βγ genes are essential for late gene expression, but dispensable for early gene expression

**Fig 1:**
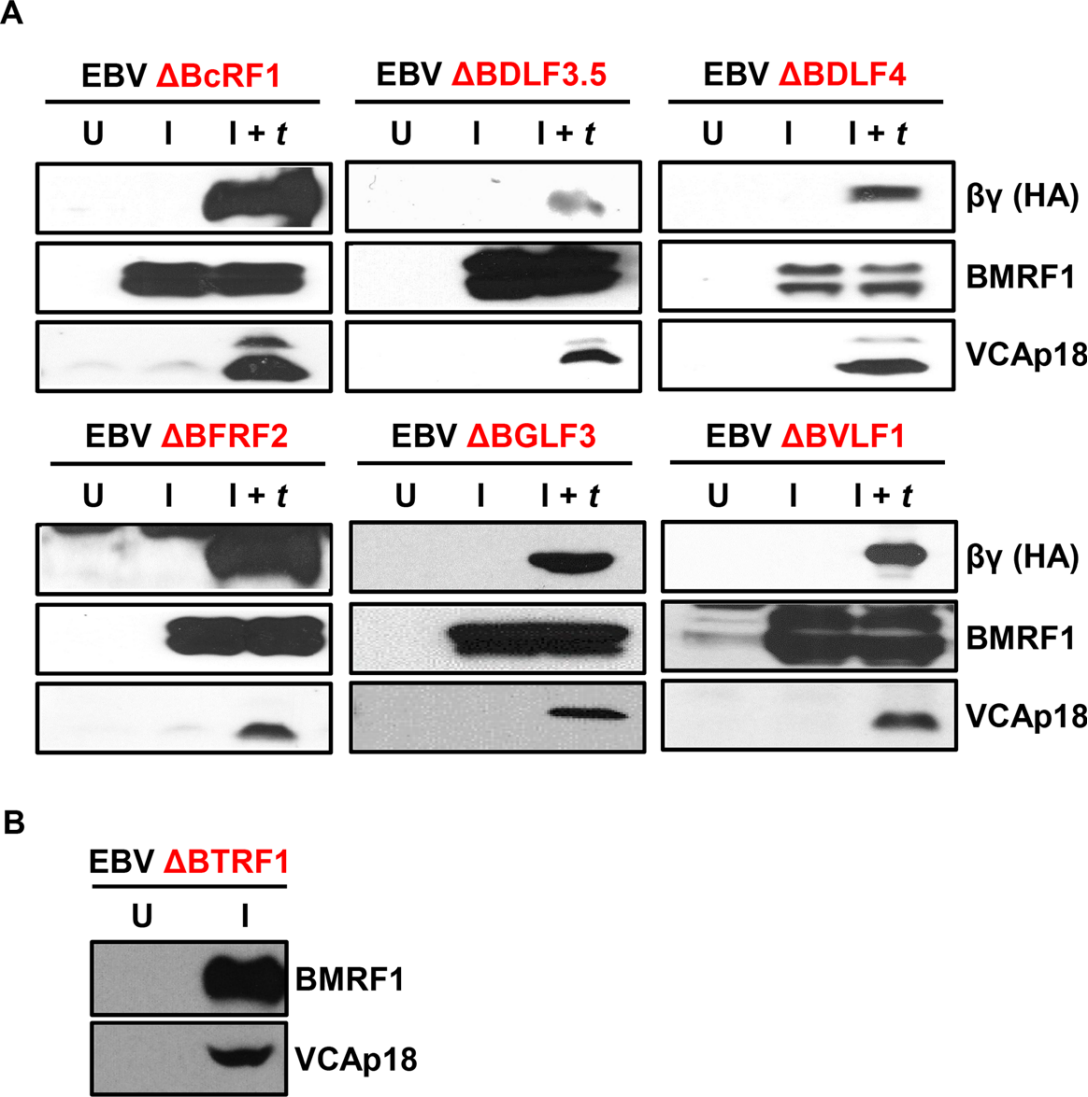
Six of the seven EBV βγ genes are essential for late gene expression. A) Immunoblots showing expression of the early gene product BMRF1 and the late gene product VCAp18 in 293 cells stably infected with EBV genomes disrupted for the indicated βγ gene under the following conditions: uninduced (U), Induced for replication by transfection with R and Z expression plasmids (I), induced for replication plus *trans*-complemented by transfection of R, Z, and an HA-tagged expression plasmid for the appropriate βγ gene (I + *t*). βγ gene expression was confirmed by HA immunoblot (top panels). Whole-cell lysates were prepared from cells at 72 hours post transfection. B) Immunoblot for BMRF1 and VCAp18, as indicated, from 293 cells stably infected with EBV ΔBTRF1 that were either uninduced (U) or induced for EBV replication (I) by transfection of R and Z expression plasmids.

To investigate the requirement of each of the 7 βγ genes for early and late gene expression, we derived 293 cell lines infected with EBV BACmids with one βγ gene mutated. We constructed the ΔBDLF3.5, ΔBGLF3, and ΔBTRF1 BACmids using the *En Passant* method [41,42] and obtained WT, ΔBcRF1 (MI-27), ΔBDLF4 (MI-84), ΔBFRF2 (MI-248) and ΔBVLF1 (MI-383) BACmids from a library of mutant EBV BACmids previously described [43]. Mutations were confirmed by sequencing of high fidelity PCR products from the appropriate region of each BACmid. Integrity of each BACmid was preliminarily assessed by restriction digestion using at least two enzymes (BamHI and EcoRI) and subsequently confirmed by *trans-*complementation (discussed below). 293 cells were infected with each EBV mutant BACmid, and several single-cell clones were selected for further validation. Each EBV Δβγ 293 line was induced for lytic replication by transfection with R and Z expression plasmids and *trans*-complemented with a plasmid expressing the missing βγ gene, where indicated. We found that 6 out of 7 βγ genes (*BcRF1*, *BDLF3*.*5*, *BDLF4*, *BFRF2*, *BGLF3*, and *BVLF1)* were required for expression of the minor capsid protein VCAp18 (product of the EBV *BFRF3* late gene) at 72 hours post induction with R and Z (Fig 1A). In contrast, EBV *BTRF1* was dispensable for VCAp18 expression (Fig 1B). None of the 7 βγ gene knockout genomes exhibited a defect in expressing the protein product of the *BMRF1* early gene (Fig 1A and 1B). These results confirm and extend previous studies that demonstrated *BcRF1*, *BFRF2*, and *BDLF4* share a common phenotype: defective late gene expression with intact early gene expression [27,36,37]. We have now shown that this same phenotype is shared by 3 other βγ genes: *BDLF3*.*5*, *BGLF3*, and *BVLF1*, but not by *BTRF1*, the last of the 7 genes conserved in all β- and γ-herpesviruses.

### βγ genes are dispensable for viral DNA replication

**Fig 2:**
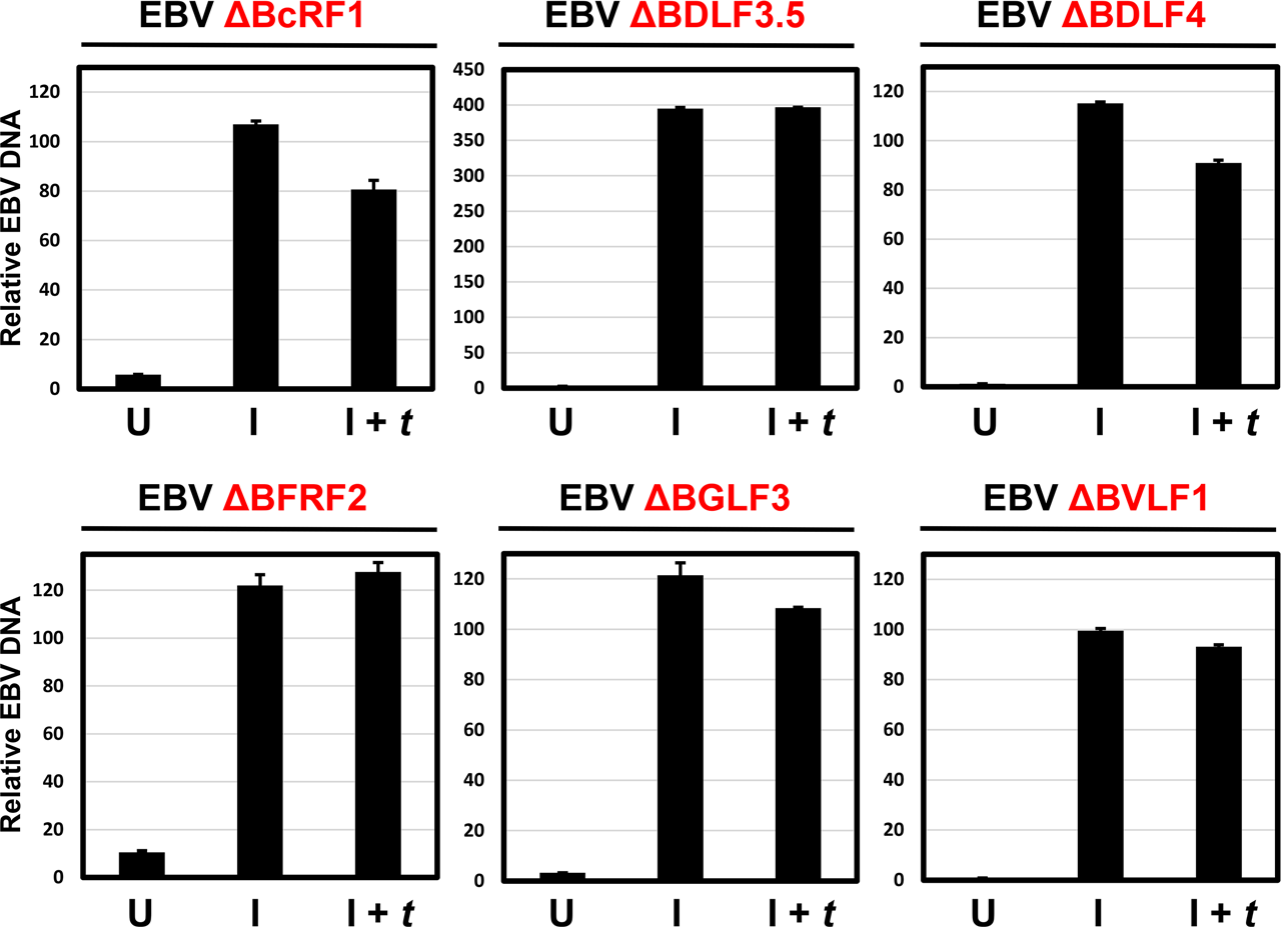
The six EBV βγ genes essential for late gene expression are dispensable for EBV lytic DNA replication. Bar plots indicating EBV DNA levels relative to cellular GADPH DNA in 293 cells stably infected with EBV BACmids disrupted for the indicated EBV βγ gene. For each cell line, genomic DNA was prepared from uninduced cells (U), cells induced by transfection with R and Z expression plasmids (I), and cells induced and *trans*-complemented by transfection with plasmids expressing R, Z, and the relevant βγ gene (I + *t*). DNA abundance was measured by qPCR using specific primers (see methods) and reported as the fold increase in the ratio of EBV/GAPDH DNA. Experiments were performed in triplicate and error bars correspond to the standard error of the mean (SEM) across the three biologic replicates.

Because late gene expression is dependent on lytic viral DNA replication, it was important to determine whether any of the βγ genes played a role in viral DNA replication. For these experiments, we induced each of the six EBV Δβγ 293 lines, which exhibited a defect in VCAp18 expression, for lytic replication by transfection of R and Z with or without βγ gene *trans*-complementation. We then measured EBV DNA (relative to cellular GAPDH) by qPCR. As demonstrated in Fig 2, a 100-fold or greater increase in EBV DNA was observed in the EBV ΔBcRF1, ΔBDLF3.5, ΔBDLF4, ΔBFRF2, ΔBGLF3, and ΔBVLF1 293 cells in response to R and Z expression. No further increase in EBV DNA was observed upon βγ *trans*-complementation, consistent with BcRF1, BDLF3.5, BDLF4, BFRF2, BGLF3, and BVLF1 playing no role in supporting EBV DNA replication. Our results are consistent with prior studies demonstrating that BALF2, BALF5, BBLF2/3, BBLF4, BMRF1, BSLF1, BKRF3, Z, R, and SM are sufficient to reconstitute EBV lytic DNA replication from a lytic origin *in vitro* [44,45]. Our results further demonstrate that none of the 6 βγ genes tested play a role in EBV lytic DNA replication that could account for their role in late gene expression.

### An EBV *Ori*Lyt *in cis* is required for activation of a TATT-containing reporter

**Fig 3:**
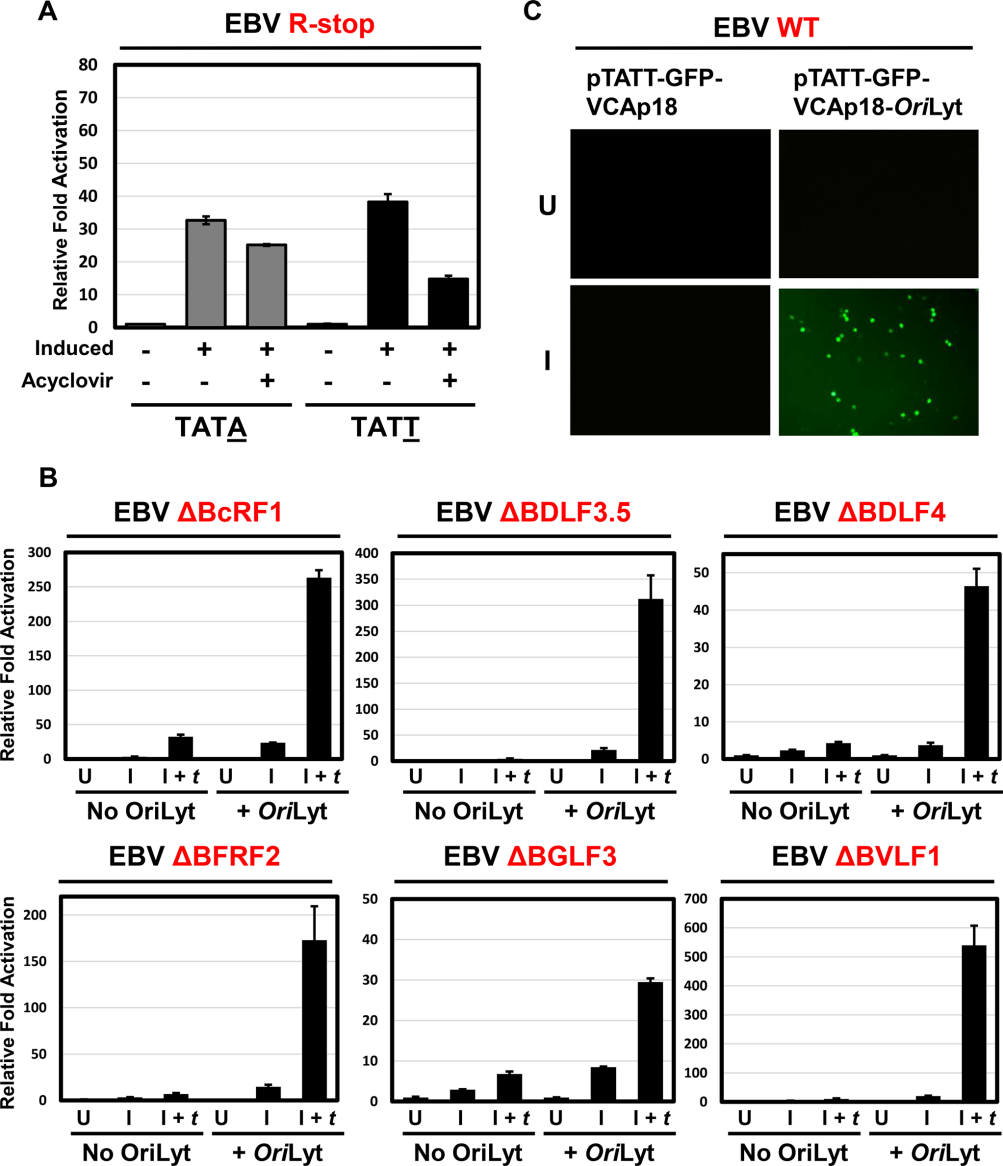
An EBV *Ori*Lyt *in cis* is required for activation of a TATT-containing reporter. A) Luciferase assays showing relative fold activation of the TATT-containing EBV *BcLF1* promoter (black bars) and its corresponding control reporter where a point mutation changes T at the fourth position to A, making a conventional TATA box (gray bars). For each condition, 293 R-stop cells were either uninduced, induced for lytic replication by transfection of R or induced for lytic replication by transfection of an R expression plasmid and also treated with the herpesvirus DNA polymerase inhibitor, acyclovir. Fold activation relative to uninduced value is reported, after normalization to renilla internal control. B) Luciferase assays showing relative fold activation of the TATT-containing EBV *BcLF1* promoter in presence or absence of EBV minimal *Ori*Lyt *in cis* in each EBV Δβγ 293 cell line. For each condition, cells were either uninduced (U), induced by transfection of R and Z (I) or induced and *trans*-complemented by transfection of R, Z, and the relevant βγ expression plasmid (I + *t*). Relative fold activation is reported after normalization to renilla internal control and to the uninduced (U) values. C) Image of 293 EBV cells transfected with either pTATT-GFP-VCAp18 (left column) or pTATT-GFP-VCAp18-*Ori*Lyt (right column) either without induction of EBV replication (top row, U) or induced by transfection of R and Z (bottom row, I). Cells were scored for GFP at 60 hours post transfection.

In order to determine whether lack of TATT promoter activation correlated with the late gene expression defect observed in our βγ gene knockout genomes, we constructed a reporter, pGL2-TATT similar to the one described by Gruffat *et al*. [27], in which the promoter of the late gene *BcLF1* (containing the non-canonical TATT) drives expression of luciferase. A corresponding control reporter, pGL2-TATA, was constructed by mutation of the fourth T to an A, resulting in a conventional TATA box. We performed reporter assays, with these reporters, using 293 cells infected with an EBV genome defective for R expression (EBV R-stop [46]) that exhibits no spontaneous lytic replication in the absence of transfected R. In this model, we observed an approximately 40-fold activation of the pGL2-TATT reporter in response to R-induced EBV replication (Fig 3A) which decreased to about 15-fold upon addition of acyclovir. Unexpectedly, the control pGL2-TATA was also activated about 35-fold, suggesting much of the observed activation was TATT independent.

We hypothesized that EBV *Ori*Lyt may be required for specific activation of TATT promoter. To test this possibility, we cloned the EBV *Ori*Lyt sequence downstream of the luciferase gene to generate pGL2-TATT-*Ori*Lyt. To evaluate the *Ori*Lyt requirement for βγ gene mediated activation of TATT-containing promoters, each EBV Δβγ 293 line was transfected with either the pGL2-TATT or pGL2-TATT-*Ori*Lyt reporter plasmids, and induced with R and Z with or without *trans*-complementation of the disrupted βγ gene. As shown in Fig 3B, the pGL2-TATT-*Ori*Lyt reporter was robustly induced (30–500 fold) in each cell line. Only weak activation of the pGL2-TATT plasmid was observed, typically at levels 1–10% of that observed for pGL2-TATT-*Ori*Lyt. Activation of the pGL2-TATT-*Ori*Lyt construct was strongly dependent on *trans-*complementation with the missing βγ gene in each cell line. Furthermore, because *Ori*Lyt-mediated DNA replication is unaffected by βγ gene *trans*-complementation (Fig 2), it is unlikely that the *Ori*Lyt dependence of the βγ gene activity is due solely to an increase in reporter copy number. Thus, 6 out of 7 EBV βγ genes (*BcRF1*, *BDLF3*.*5*, *BDLF4*, *BFRF2*, *BGLF3*, and *BVLF1*) contribute to activation of a TATT reporter plasmid and the presence of the EBV *Ori*Lyt is required for high-level activation.

We also examined the requirement for *Ori*Lyt from a different late promoter using the pTATT-GFP-VCAp18-*Ori*Lyt and pTATT-GFP-VCAp18 reporter plasmids which express a GFP-VCAp18 fusion protein from the native VCAp18 (*BFRF3*) promoter with or without the EBV *Ori*Lyt sequence cloned 3’ to the expression cassette, respectively. Both plasmids have an EBV *Ori*P latent origin, permitting their maintenance as an episome in EBV-infected cells. Initially these reporters were transfected into EBV WT 293 cells with or without R and Z expression plasmids to induce lytic replication. Cells were subjected to microscopy at 24, 48, and 60 hours after transfection to test for expression of GFP-VCAp18. As shown in Fig 3C, GFP-VCAp18 expression was only observed at 60 hours post-induction when the transfected construct contained EBV *Ori*Lyt (bottom right panel). No GFP-positive cells were scored in uninduced cells or at 24 or 48 hours post lytic induction, consistent with the late kinetics of the *BFRF3* promoter.

**Fig 4:**
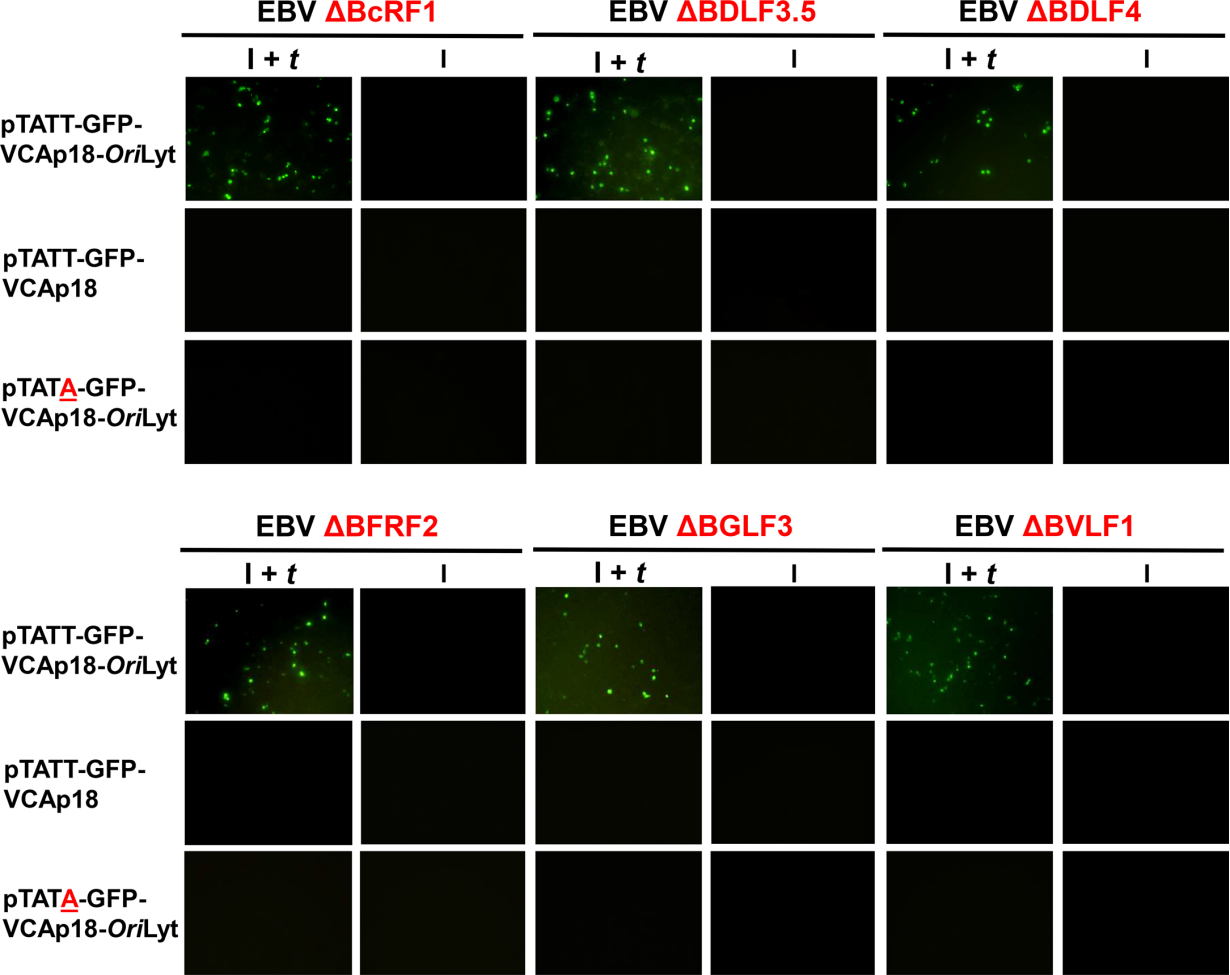
Loss of the TATT motif or *Ori*Lyt disrupts βγ gene dependent transcription. Images of 293 cells infected with the indicated EBV βγ gene deletion mutant and transfected with either pTATT-GFP-VCAp18-*Ori*Lyt (top rows), pTATT-GFP-VCAp18 (middle rows) or pTATA-GFP-VCAp18-*Ori*Lyt (bottom rows) either induced (I) by transfection of R and Z (second, forth, and sixth columns) or induced and *trans-*complemented (I + *t*) by transfection of R, Z, and the relevant βγ expression plasmid (first, third, and fifth columns). Cells were scored for GFP at 60 hours post transfection.

We further evaluated the requirements of βγ gene mediated late gene transcription by constructing an additional reporter, pTATA-GFP-VCAp18-*Ori*Lyt, in which the *BFRF3* promoter TATT sequence was mutated to the canonical TATA. As shown in Fig 4, 293 cells infected with EBV ΔBcRF1 only expressed the GFP-VCAp18 fusion protein in cells induced for replication, when *trans*-complemented with a BcRF1 expression plasmid (I + *t* condition). Further, BcRF1-mediated transcription was not supported by reporters lacking *Ori*Lyt (middle panels) or bearing the TATT to TATA mutation (bottom panels). Results using 293 cells infected with EBV ΔBDLF3.5, EBV ΔBDLF4, EBV ΔBFRF2, EBV ΔBGLF3, and EBV ΔBVLF1 were similar: GFP-VCAp18 expression was only observed from the pTATT-GFP-VCAp18-*Ori*Lyt reporter and required induction of viral replication plus *trans*-complementation of the absent βγ gene. Collectively these results suggest that late gene promoter activation requires each of 6 βγ genes, the non-canonical TATT-box, and the EBV origin of lytic replication.

### Construction of an EBV mutant BACmid defective for lytic viral DNA replication

**Fig 5:**
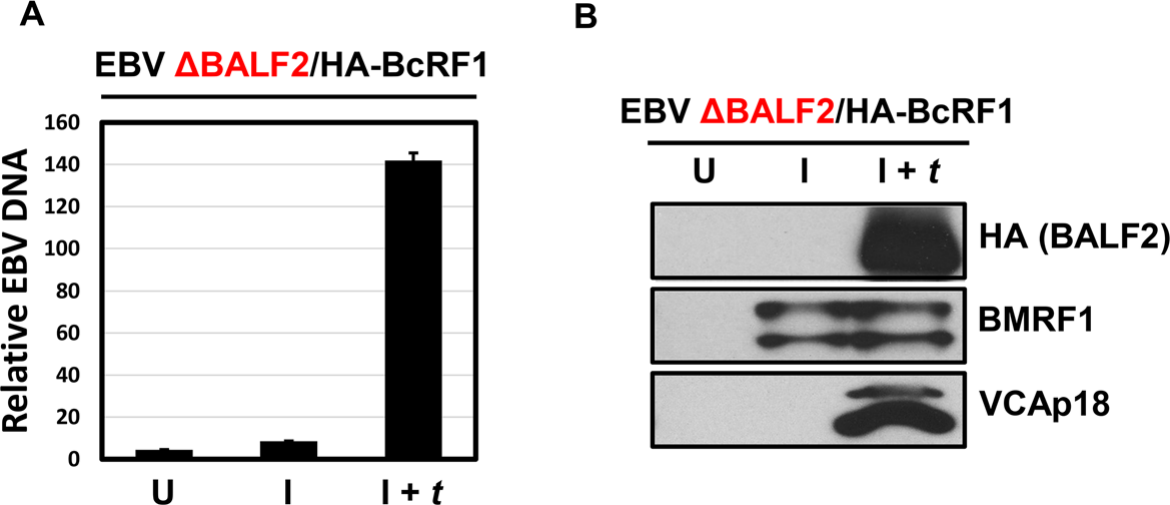
A BALF2 null EBV genome is defective for lytic DNA replication and late gene expression. A) Bar plot indicating relative EBV DNA levels in 293 cells stably infected with the EBV ΔBALF2/HA-BcRF1 BACmid. Genomic DNA was prepared from uninduced cells (U), cells induced by transfection with R and Z expression plasmids (I), and cells induced and *trans*-complemented by transfection with R, Z, and BALF2 expression plasmids (I + *t*). DNA abundance was measured by qPCR using specific primers (see methods) and reported as the fold increase in the ratio of EBV/GAPDH DNA. Experiments were performed in triplicates and error bars correspond to the SEM across the three biologic replicates. B) Immunoblots showing expression of the early gene product BMRF1 and the late gene product VCAp18 in 293 cells stably infected with the EBV ΔBALF2/HA-BcRF1 BACmid under the following conditions: uninduced (U), Induced for replication by transfection of R and Z (I), induced for replication and *trans*-complemented by transfection of R, Z, and an HA-tagged BALF2 (I + *t*). BALF2 expression was confirmed by HA immunoblot (top panel). Whole cell lysates were prepared from cells at 72 hours post transfection.

Because the EBV *Ori*Lyt functions as both a replication origin and a transcriptional enhancer, it was important to determine whether or not the requirement for *Ori*Lyt on the reporter reflected a need for the reporter plasmid DNA to be replicated in order to activate the TATT element. To assess this requirement for newly replicated DNA, we constructed a DNA replication-defective EBV BACmid by inserting a stop codon in place of the second amino acid codon of the *BALF2* gene which encodes the single stranded DNA binding protein, an essential component of the viral DNA replication machinery. This BACmid had previously been modified by insertion of a sequence encoding an N-terminal HA epitope tag upstream of the *BcRF1* gene and therefore was designated as EBV ΔBALF2/HA-BcRF1. 293 cells were stably infected with EBV ΔBALF2/HA-BcRF1, then induced with R and Z and *trans*-complemented with a plasmid expressing BALF2, where indicated. Cells were harvested 48 hours post induction and qPCR was performed with primers specific to EBV *Ori*Lyt and the cellular GAPDH. As shown in Fig 5A, when the *BALF2* gene is mutated, viral DNA replication does not occur. This defect is rescued when BALF2 is expressed *in trans*. Immunoblotting revealed that EBV ΔBALF2/HA-BcRF1 239 cells express the BMRF1 early gene product in the absence of BALF2, but that expression of the VCAp18 late gene product is strictly dependent on BALF2 *trans*-complementation (Fig 5B). Furthermore, loss of BALF2 did not have an effect on relative βγ mRNA levels (S1 Fig). The replication-defective EBV ΔBALF2/HA-BcRF1 293 cells, therefore, provide an appropriate system for assessing the requirement of viral DNA synthesis for late gene expression, while bypassing the potential cytotoxic and/or off-target effects of the herpesvirus DNA polymerase inhibitor, acyclovir.

### Viral DNA replication is necessary for TATT promoter activation

**Fig 6:**
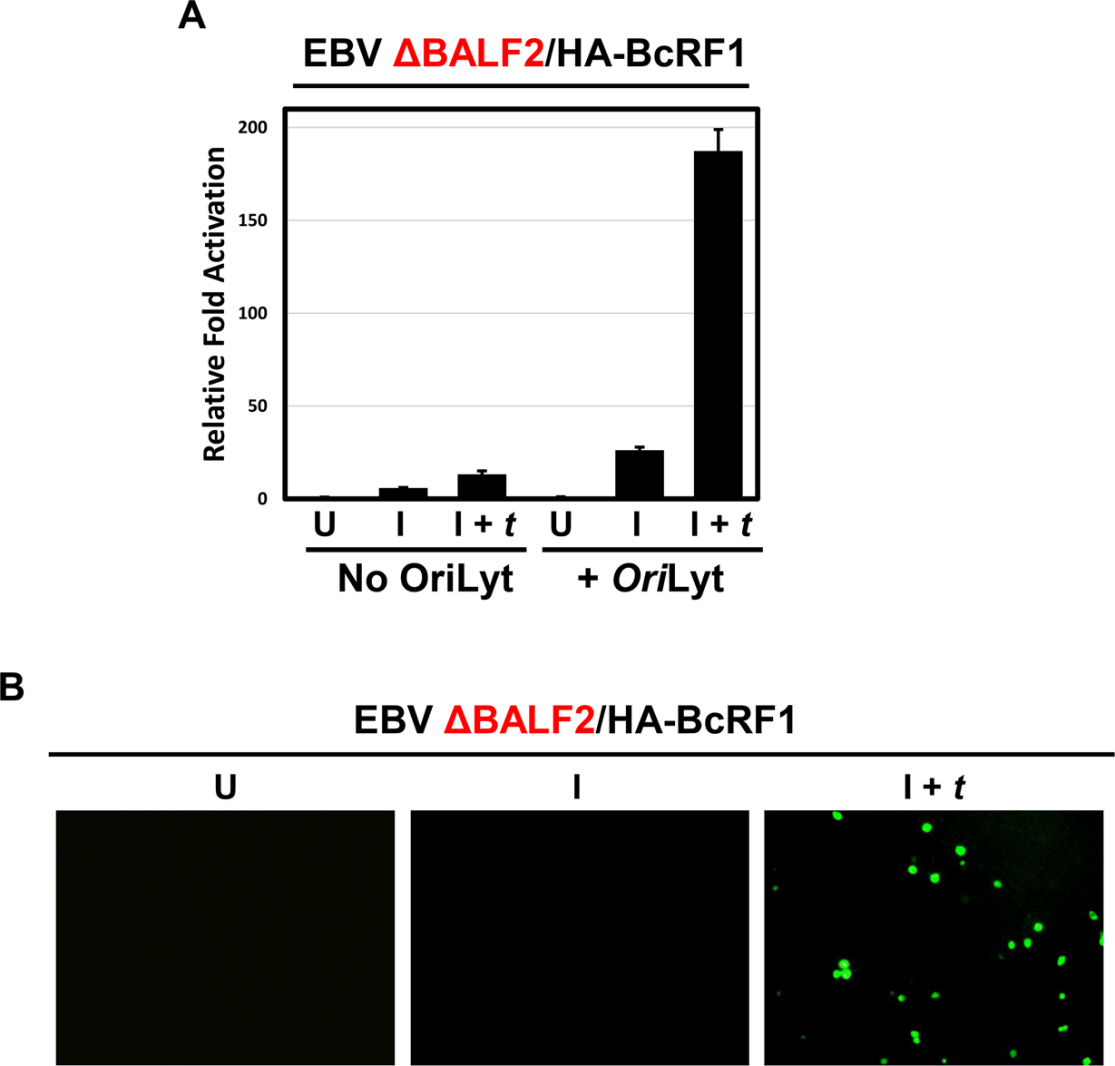
EBV lytic DNA replication is necessary for TATT reporter activation. A) Luciferase assay showing relative fold activation of the TATT-containing EBV *BcLF1* promoter in presence or absence of EBV minimal *Ori*Lyt *in cis* in the replication defective EBV ΔBALF2/HA-BcRF1 293 cells. For each condition, cells were either uninduced (U), induced by transfection of R and Z (I) or induced and *trans*-complemented by transfection of R, Z, and BALF2 (I + *t*). Relative fold activation is reported after normalization to renilla internal control and to the uninduced (U) values. B) pTATT-GFP-VCAp18-*Ori*Lyt, expressing the fusion protein GFP-VCAp18 under control of the late VCAp18 promoter (i.e. BFRF3p) and containing EBV *Ori*Lyt was transfected in 293 cells containing EBV ΔBALF2/HA-BcRF1 genome. Cells were either uninduced (U), induced by transfection of R and Z (I) or induced and *trans*-complemented by transfection of R, Z and BALF2 (I + *t*). Cell were scored for GFP at 60 hours post transfection.

To understand the role of viral DNA replication for late gene expression, we measured TATT promoter activity in 293 cells containing the replication-defective EBV BACmid described in Fig 5. EBV ΔBALF2/HA-BcRF1 293 cells were transfected with the pGL2-TATT and pGL2-TATT-*Ori*Lyt (containing the promoter of the late gene *BcLF1*) reporter plasmids and induced with R and Z in presence or absence of BALF2 *trans-*complementation. Cells were harvested 48 hours post lytic induction and reporter assays were performed. As shown in Fig 6A, the TATT promoter can only be robustly activated when the EBV *Ori*Lyt is present on the plasmid and viral DNA replication is rescued via BALF2 *trans*-complementation. This is consistent with the requirement of *Ori*Lyt-mediated viral DNA synthesis for TATT promoter activation.

To further confirm the requirement of viral DNA synthesis for TATT activation and late gene expression, we examined expression from the pTATT-GFP-VCAp18-*Ori*Lyt reporter in the DNA replication-defective EBV ΔBALF2/HA-BcRF1 infected 293 cells. Cells were induced with R and Z, *trans*-complemented with a plasmid expressing BALF2 to rescue the replication defect where indicated, and assessed for GFP positivity at 24, 48, and 60 hours post induction by microscopy. GFP-positive cells were only observed at 60 hours when viral DNA replication was rescued by BALF2 *trans*-complementation (Fig 6B). No GFP-positive cells were observed at 24 or 48 hours post induction, consistent with late kinetics of GFP-VCAp18 expression. These observations are in accord with the hypotheses that *Ori*Lyt-mediated DNA replication from the pTATT-GFP-VCAp18-*Ori*Lyt reporter is required for GFP-VCAp18 expression to occur.

### EBV TATT-driven late gene expression requires *Ori*Lyt *in cis*

**Fig 7:**
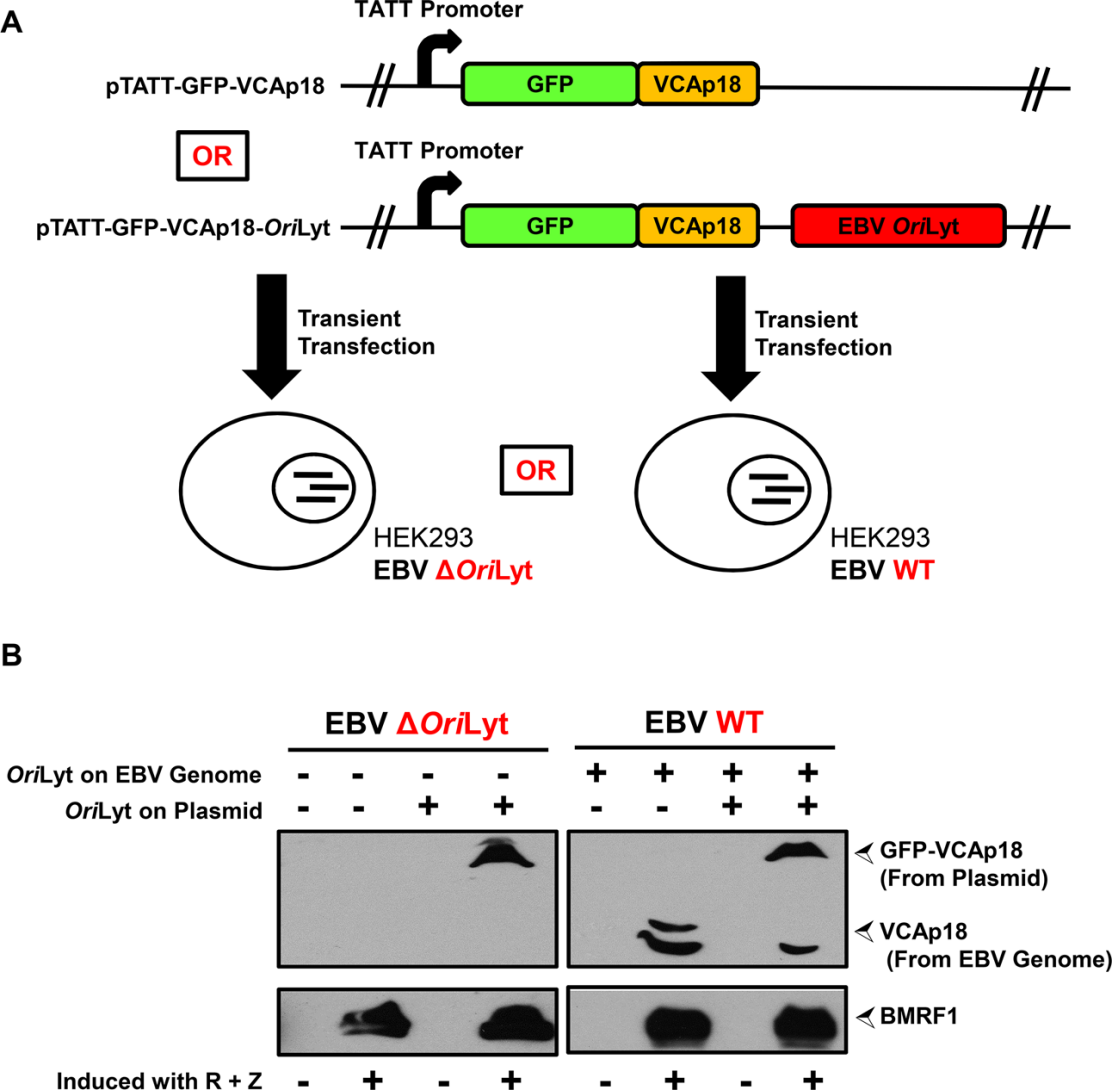
EBV *Ori*Lyt is required *in cis* for late gene expression in the context of the intact viral genome. A) Schematic of the experiment shown in Fig 7B, in which 293 cells infected with either a wildtype EBV genome (EBV WT) or an EBV genome lacking an *Ori*Lyt (EBV Δ*Ori*Lyt) are transfected with either a plasmid that expresses a GFP-VCAp18 fusion protein under the control of the native VCAp18 promoter (i.e., *BFRF3*p) or an identical plasmid that also contains the EBV *Ori*Lyt. B) Immunoblots showing expression of the early gene product BMRF1 (bottom panels) and the late gene products GFP-VCAp18 (from plasmid) and VCAp18 (from EBV genome) (top panels) in presence or absence of EBV *Ori*Lyt on either the plasmid or the intact EBV genome. pTATT-GFP-VCAp18 or pTATT-GFP-VCAp18-*Ori*Lyt were transfected into cells stably infected with either EBV Δ*Ori*Lyt (left panels) or EBV WT (right panels) genomes. For each condition, cells were either uninduced or induced by transfection of R and Z expression plasmids. Immunoblots were performed at 60 hours post-transfection.

Although we found a dependence on *Ori*Lyt-mediated DNA replication in two distinct reporter systems, it was important to determine if this was required for late gene expression in the context of the EBV genome. Therefore, we first deleted the remaining *Ori*Lyt from the EBV WT BACmid (one *Ori*Lyt is absent because the BACmid is derived from the B95.8 EBV strain) and established stable 293 cell lines. pTATT-GFP-VCAp18-*Ori*Lyt or pTATT-GFP-VCAp18 were transfected into 293 cells stably infected with EBV WT or EBV Δ*Ori*Lyt and the lytic cycle was induced by transfection of R and Z expression plasmids (Fig 7A). We then performed immunoblot assays to measure VCAp18 from the endogenous EBV genome or GFP-VCAp18 from the exogenous plasmid in the presence or absence of an *Ori*Lyt *in cis*. Cells were harvested 60 hours post induction and immunoblot assays were performed using antibodies against the early protein BMRF1 and the late protein VCAp18. VCAp18 signal from the endogenous EBV genome was detected in the context of WT EBV, but not with EBV Δ*Ori*Lyt genome even when *Ori*Lyt was present *in trans* on the pTATT-GFP-VCAp18-*Ori*Lyt plasmid (Fig 7B). Likewise, GFP-VCAp18 was expressed from pTATT-GFP-VCAp18-*Ori*Lyt but not from pTATT-GFP-VCAp18, even when an *Ori*Lyt was present on the WT EBV genome *in trans*. Thus, the late protein VCAp18 is only expressed when *Ori*Lyt is present *in cis*, but the early protein BMRF1 is expressed equally well in the presence or absence of *Ori*Lyt. Thus, we conclude that *Ori*Lyt-mediated DNA replication is specifically required *in cis* for EBV late gene expression from TATT promoters.

### The BcLF1 late mRNAs co-localize exclusively with EBV DNA replication factories

**Fig 8:**
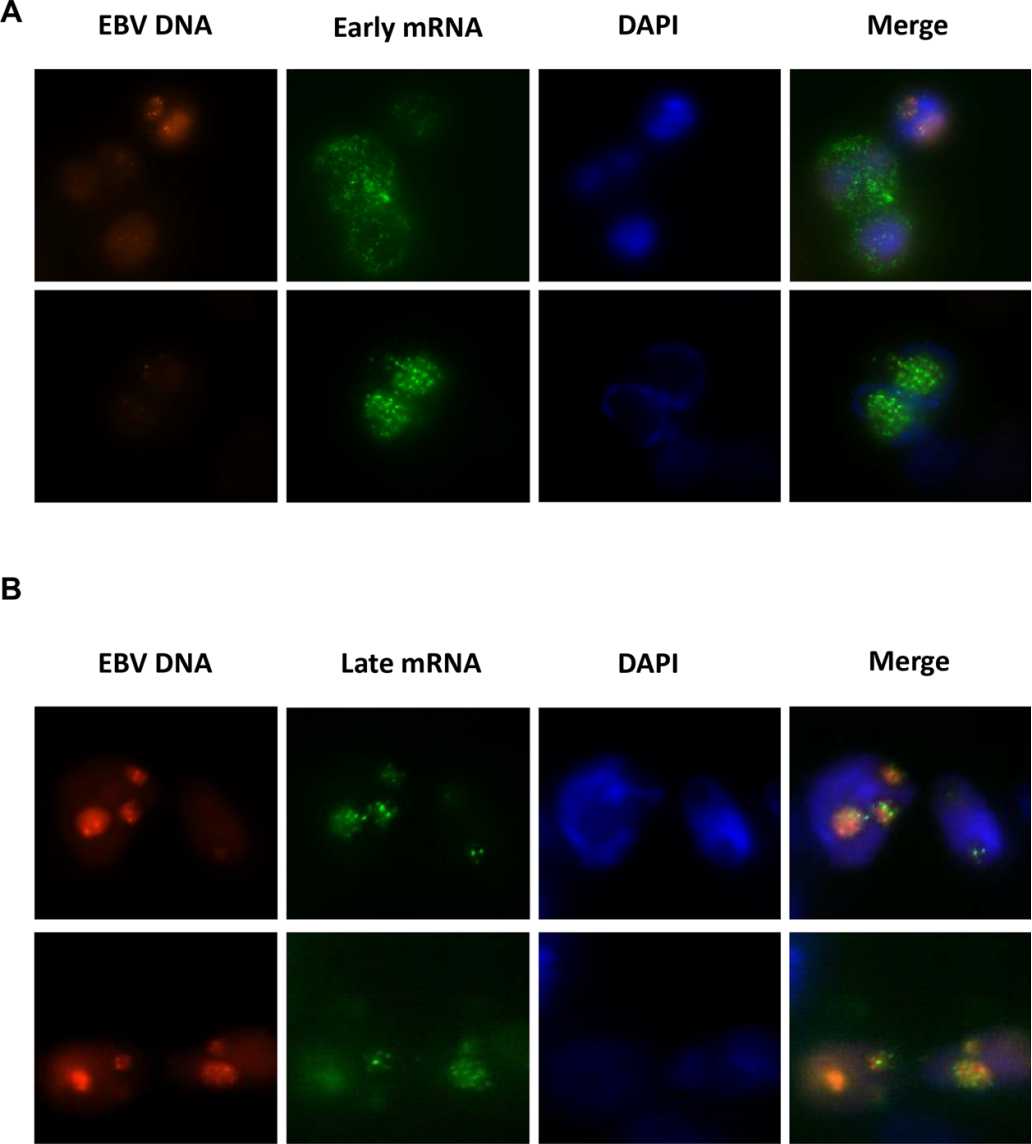
Late gene mRNAs co-localize exclusively with sites of EBV DNA replication. A) Fluorescent images of 293 cells infected with the “visible” EBV derivative at 36 hours post induction of replication. Early DNA replication factories are present in some cells (red), FISH for the early BALF2 mRNA (green) reveals punctate staining with both nuclear and cytoplasmic localization, and nuclei are visualized by DAPI staining (blue). B) Images of the same 293 cells infected with visible EBV are shown 48 hours post induction. EBV DNA replication factories are visible as discrete nuclear zones due to binding of the LacI-tdTomato fusions to EBV DNA (red), and FISH for the late BcLF1 mRNA (green) reveals punctate foci which co-localize with DNA replication factories (merge).

It has been previously shown that EBV lytic DNA replication occurs in nuclear compartments or “factories” that are devoid of histones and cellular DNA [47]. The requirement for an *Ori*Lyt *in cis* suggests a functional link between these factories and late gene transcription. Using a previously described “visible” EBV derivative that contains 250 lacO binding sites and expresses a LacI-tdTomato fluorescent fusion protein, we identified DNA replication factories and sought to determine if they co-localized with late mRNAs. When cells were imaged at 36 hours, early DNA replication factories were observed in some cells (Fig 8A). In these cells, fluorescent in situ hybridization (FISH) for the early BALF2 mRNA revealed both a nuclear and cytoplasmic distribution. At 48 hours post-induction, EBV DNA replication factories were evident as large foci of red fluorescence (Fig 8B, Visible EBV panels). Detection of the BcLF1 mRNA at this time (Fig 8B, late mRNA panels) by FISH showed punctate staining that co-localized with sites of DNA replication (Fig 8B, merge). These results demonstrate that late mRNAs synthesis corresponds temporally and geographically with sites of productive EBV DNA synthesis.

## Discussion

**Fig 9:**
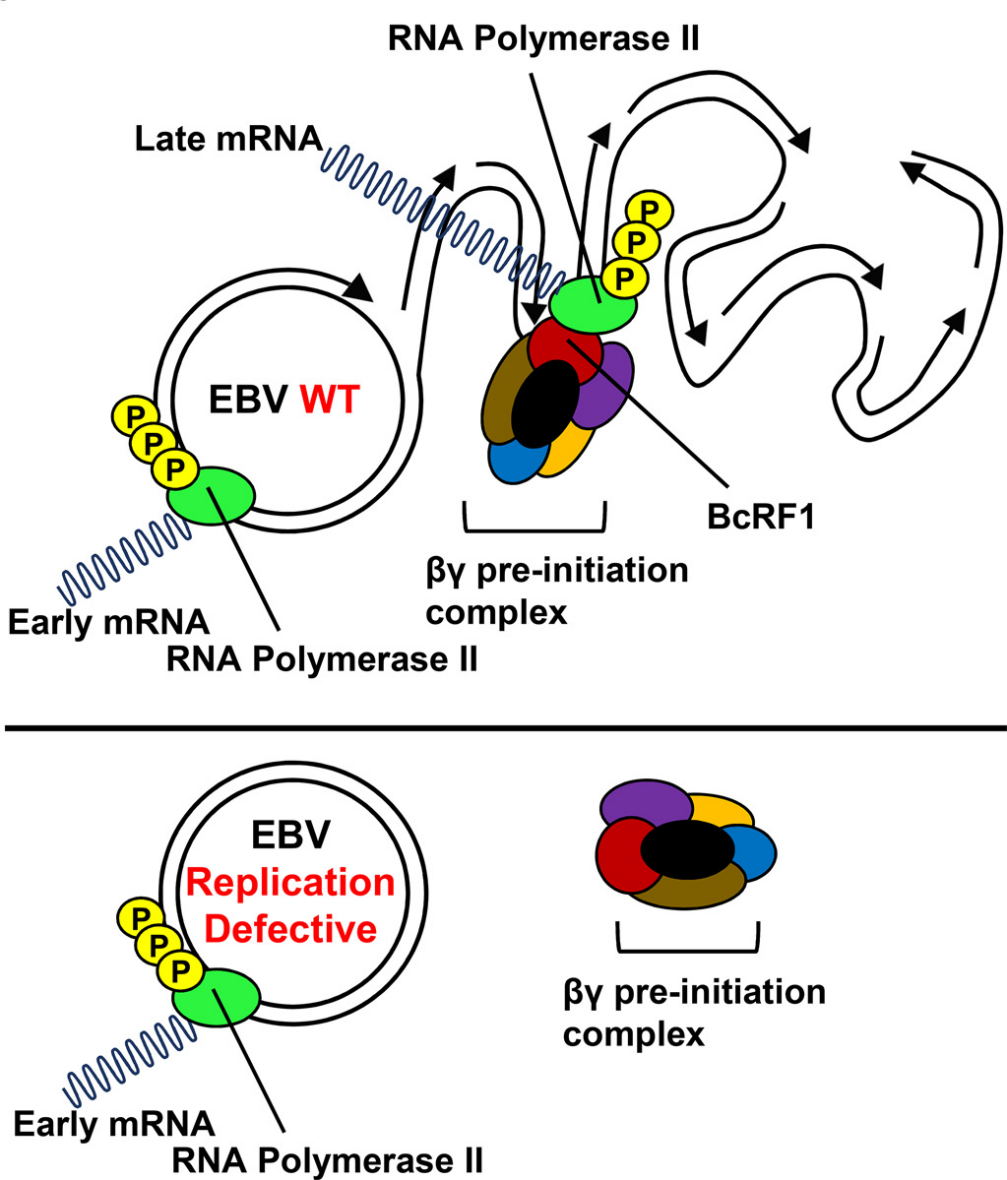
Model for EBV late gene expression from the newly replicated DNA. Model indicating transcription of late genes from the newly replicated viral DNA via six gene products conserved in β- and γ-herpesviruses (βγ genes). BcRF1, the EBV TATT-binding protein, directly binds the non-canonical TATT sequence found in the late gene promoters. According to this model, the parental EBV DNA is not suitable for binding of the viral pre-initiation complex encoded by the βγ genes and thus, late genes cannot be expressed from replication-defective genomes.

A growing body of evidence supports the hypothesis that β- and γ-herpesviruses control late gene expression via a virus-encoded pre-initiation complex [26–37]. Here, we analyzed the phenotype of EBV mutants with disruptions in each of the seven genes with homologs in β- and γ-herpesviruses (βγ genes). We found that 6 of the 7 βγ genes are essential for expression of the EBV VCAp18 late gene product and dispensable for lytic DNA replication (Figs 1 and 2). These results are consistent with recently published reports assessing the roles of the *BcRF1*, *BFRF2*, and *BDLF4* genes [27,36,37]. We extended these findings, demonstrating here that this phenotype is also true for three additional EBV βγ genes (*BDLF3*.*5*, *BGLF3*, and *BVLF1)*, but not for the last EBV βγ gene, *BTRF1*. These same six proteins, when exogenously expressed in EBV-negative 293 cells, have been reported to form a viral pre-initiation complex that mediates transcription from the non-canonical TATT box found in late gene promoters [37]. Unexpectedly, using 293 cells infected with EBV genomes deleted for specific βγ genes, we found that βγ gene-mediated activation of a TATT reporter was weak and non-specific (relative to TATA) unless an EBV *Ori*Lyt was present on the reporter (Figs 3 and 4). Although the EBV *Ori*Lyt acts as both an enhancer and an origin of DNA replication, we demonstrated that the TATT-*Ori*Lyt reporter could not be activated in 293 cells infected with a replication defective EBV genome. Thus, lytic DNA replication is required for optimal EBV-mediated activation of the TATT reporter. We further demonstrated, using a combination of *Ori*Lyt deleted EBV genomes and plasmids, that an *Ori*Lyt *in cis* is essential for TATT-driven late gene expression (Fig 7). Finally, we demonstrated that the late BcLF1 mRNA exclusively localizes to the EBV replication factories; In contrast, the early BALF2 mRNA is more broadly distributed (Fig 8). Based on these results we propose a model where βγ gene products mediate recruitment of RNA polymerase II to transcribe EBV late genes from newly replicated viral DNA (illustrated in Fig 9).

In an effort to most closely model EBV late gene expression, our experiments presented here were performed in cells infected with EBV WT or EBV mutant genomes. This approach offers multiple advantages over reporter assays in EBV-negative cells. First, EBV lytic gene products required for late gene expression are expressed under the control of the native promoters assuring proper temporal kinetics and levels of expression. Additionally, because the extent to which βγ gene products are modulated by other EBV lytic proteins (e.g., the BGLF4 kinase) remains to be determined, it is preferable to study their role in the presence of the EBV genome. Finally, late gene expression via βγ gene products has been compared to the T4 bacteriophage late gene regulation [34]. It is interesting to note that for T4, DNA nicks bypass the dependence of late gene expression on DNA replication [48]. If this holds true for EBV, one would expect the apparent requirement for *Ori*Lyt in reporter assays to be strongly influenced by the transfection conditions and the integrity of the reporter plasmid. For these reasons, we have endeavored to make or confirm all the observations reported here in EBV infected cells with plasmids that can be maintained extrachromosomally.

Why would an *Ori*Lyt *in cis* be required for late gene mediated transcriptional activation? The full EBV *Ori*Lyt is both a strong enhancer and a DNA replication origin. Indeed, transcriptional activation and the subsequent formation of RNA-DNA hybrids at *Ori*Lyt appear to be essential for initiation of lytic DNA replication in γ-herpesviruses (as well as cytomegalovirus) which lack dedicated origin binding proteins [49,50]. Because a minimal *Ori*Lyt exhibiting 1% of the DNA replication activity of full *Ori*Lyt supports late gene expression [40], it has been suggested that other mechanisms such as *Ori*Lyt dependent PML body association may be important [51]. Our results do not exclude these additional mechanisms; however, our ΔBALF2 experiment (Fig 6) and the ability of viral DNA polymerase inhibitors to block late gene expression argue that the requirement for *Ori*Lyt includes its DNA replication function. Further, we believe the linkage of βγ mediated late gene transcription to the requirement for an *Ori*Lyt *in cis*, strongly implies that the parental EBV genome is not competent for late gene transcription until it has been replicated.

In EBV, the viral replication machinery consisting of the BALF5 DNA polymerase, BMRF1 DNA polymerase processivity factor, BALF2 single-stranded DNA binding protein, and the BBLF4-BSLF1-BBLF2/3 helicase-primase complex replicate the EBV genome during productive infection in discrete nuclear sites called factories. Replication factories form as a consequence of a dramatic rearrangement in nuclear architecture, in which the cell DNA moves to the nuclear periphery and the nucleus becomes dominated by the expanding replication factory. These factories are characterized by the presence of newly synthesized viral DNA and the EBV DNA polymerase and its processivity factor, BALF5 and BMRF1 respectively, but are devoid of cellular DNA, histones, and PCNA [47]. Other investigators have used BMRF1 immunofluorescence to define EBV replication factories as BMRF1 cores [52,53] that lend additional support to our model that late gene mRNAs are transcribed from newly replicated viral DNA. Using pulse-chase experiments, Sugimoto *et al*. demonstrated that newly synthesized viral genomes organized around the BMRF1 cores were transferred inward, leaving the parental template outside the BMRF1 cores [53]. Using a “visible” EBV derivative, we demonstrated that the BcLF1 late mRNA co-localizes exclusively with DNA replication factories, in contrast the BALF2 early mRNA which was predominantly cytoplasmic at the time points studied and often identified in cells that had not formed DNA replication factories. Interestingly, BcRF1, the EBV TATT-binding protein, is primarily localized inside the BMRF1 cores where newly replicated DNA is localized [54], and therefore is well positioned to interact with newly synthesized viral DNA. Although not definitively demonstrated, these findings collectively suggest that EBV, and by analogy other β- and γ- herpesviruses, employ a set of evolutionary conserved proteins to mediate transcription of late genes from the newly replicated DNA template.

Although the dependence of late gene expression on viral DNA replication is observed in all herpesviruses, our work adds to an increasing body of evidence that β- and γ- herpesviruses regulate their late genes by a distinct mechanism. α-herpesvirus late genes are similar to β- and γ-herpesvirus late genes in that their expression is controlled by a short proximal TATA box [55], but differ in that this sequence neither appears to be similar to the unconventional TATT box found in β- and γ-herpesviruses, nor is it recognized by unique virally encoded trans-acting factors. In herpes simplex virus (HSV), a limited number of genes such as ICP4, ICP8 and ICP27 have been implicated [56–64], but their disruption impairs other stages of HSV replication as well as late gene expression. The preponderance of evidence suggests that β- and γ-herpesviruses encode six conserved gene products that mediate late gene expression by recruiting RNA polymerase II to TATT elements in late gene promoters. Our demonstration that *Ori*Lyt is required *in cis* is particularly appealing as it further explains the dependence of late gene expression on lytic DNA replication. That α-herpesviruses recapitulate this phenotype with a mechanistically distinct regulatory apparatus suggests that strong convergent evolutionary pressure exists to regulate late gene expression in a DNA replication-dependent fashion.

What are the potential evolutionary advantages of a viral pre-initiation complex linking late gene expression to DNA replication? One possibility is that such a system provides direct means for expressing structural protein mRNAs in direct proportion to the quantity of replicated viral DNA available for packaging. Such a system may also contribute to viral immune evasion by ensuring that regardless of the transcription factor milieu of the infected cell, a large number of potential antigens cannot be expressed unless the virus has committed to replicating its DNA. In a similar vein, by obviating the need for cell transcription factors to promote late gene expression, there is less potential for interference or cross-talk among viral lytic promoters. Given the propensity of α-herpesviruses to establish latency in neurons, it is tempting to speculate that some or all of these advantages facilitated the success of β- and γ-herpesviruses in establishing latency in cells with potential for dynamic changes in transcriptional regulation such as lymphocytes and hematopoietic stem cells.

If indeed β- and γ-herpesviruses direct RNA polymerase II to transcribe newly replicated DNA, it raises many interesting questions since mRNA transcription is normally suppressed during DNA replication. It is likely that the βγ pre-initiation complex must overcome a number of cellular checkpoints designed to prevent such transcription. Because nascent viral DNA is thought to be devoid of histones [47], proteins that bind specific histone modifications would need to be recruited by other means. Some progress has been made in mapping the protein-protein interactions that allow the βγ gene products to assemble into a pre-initiation complex [65,66]. At present, we only understand the specific role of one of these βγ genes, the TBP homolog-encoded *BcRF1*. The BDLF4 gene product and its paralogs are notable for a probable zinc coordination motif (Cys-X_2_-Cys-X_3_-His-X-Cys-X_5,6_-Cys-X_10_-Cys) in their N-termini that could mediate DNA or protein interactions. It will be important to determine the functions of the remaining βγ gene products and understand how they act to permit transcription from this atypical DNA template.

## Materials and Methods

### Cell lines and culture

All cell lines were maintained in Dulbecco’s modified Eagle medium (DMEM) supplemented with 10% fetal bovine serum (FBS) and 1% penicillin-streptomycin. EBV-negative 293 cell lines were obtained from Bill Sugden, University of Wisconsin-Madison. 293 cells infected with the EBV R-stop mutant (a gift from Shannon Kenney, University of Wisconsin-Madison) have been previously described [46].

### Construction of EBV mutant genomes

The EBV p2089 BACmid contains the complete genome of the B95.8 strain of EBV in addition to a cassette containing the prokaryotic F-factor as well as the green fluorescent protein (*GFP*) and *Hygromycin B* resistance genes in the B95.8 deletion as previously described [67]. The EBV Wild-Type (WT) BACmid used in these studies is a modification of the p2089 BACmid lacking a functional *GFP* ORF. The WT, ΔBcRF1 (MI-27), ΔBDLF4 (MI-84), ΔBFRF2 (MI-248), and ΔBVLF1 (MI-383) BACmids (Ya-Fang Chiu at the laboratory of Shih-Tung Liu) were part of a comprehensive library of mutant EBV genomes and have been previously described [43]. This same WT GFP-negative p2089 BACmid is the parental BACmid to all mutants in this study. All remaining mutant BACmids were constructed using the GS1783 *E*. *coli*–based *En Passant* method previously described [41,42]. ΔBALF2/HA-BcRF1 BACmid is a double-mutant, in which the *BcRF1* gene is N-terminally tagged with HA and the gene encoding the single-stranded DNA binding protein BALF2 is disrupted by a single nucleotide mutation changing the second amino acid residue in the *BALF2* ORF to a stop codon. Similarly, ΔBDLF3.5 and ΔBGLF3 BACmids were constructed by introducing stop codons via point mutations in place of the amino acid residues 9 and 6 respectively. The ΔBTRF1 BACmid was constructed by introducing stop codons at amino acid residues 6 and 8, in addition to insertion of a *Kanamycin* resistance gene in the *BTRF1* ORF (between coordinates 127,460 and 127,461; AJ507799.2). The Δ*Ori*Lyt BACmid was constructed by replacing the EBV *Ori*Lyt (coordinates 37,978–42,663; AJ507799.2) with a *Kanamycin* resistance gene. Finally, the *Chloramphenicol* cassette in the F-factor of each WT, ΔBALF2/HA-BcRF1, ΔBDLF3.5, ΔBGLF3 BACmids was replaced with *Kanamycin*. Kanamycin resistance facilitated the transfer of all BACmids to the chloramphenicol-resistant BM2710 *E*. *coli* used for infection of 293 cells. The integrity of all BACmids was confirmed by analyzing the restriction digestion patterns with multiple enzymes. Furthermore, all mutations were confirmed by high fidelity PCR amplification and sequencing the mutated junctions. The comprehensive list of primers used for generation and confirmation of all mutants is found in Tables 1 and 2 respectively.

**Table 1:** Primers used for construction of EBV mutant BACmids.

Primer Name	Sequence	Purpose
ΔBALF2-Fwd	TGTGGGACTGGGAGGCCGGGGCGATACCTTGGGCATCATGTAGGGTGCACTGACTAGCGAGGATAATCTGGAGGATGACGACGATAAGTAGGG	ΔBALF2/HA-BcRF1
ΔBALF2-Rev	ACCCGGCTGGCTCTGGCTGCCCAGATTATCCTCGCTAGTCAGTGCACCCTACATGATGCCCAAGGTATCGCCAACCAATTAACCAATTCTGATTAG	ΔBALF2/HA-BcRF1
HA-BcRF1-Fwd	GGGGAAGGGTCTGTTTTCCACACCCTCATTTGAGGCCATGACATATCCATATGACGTTCCAGATTACGCTACACAAGGAGGATGACGACGATAAGTAGGG	ΔBALF2/HA-BcRF1
HA-BcRF1-Rev	AGAAGCCCTCGAGACCTCCCCCCATCTCCCTCTTACCTTGTGTAGCGTAATCTGGAACGTCATATGGATATGTCATGGCAACCAATTAACCAATTCTGATTAG	ΔBALF2/HA-BcRF1
ΔBDLF3.5-Fwd	ACAAACAAGAGGTGAAATGTCTGCCCCCGGATGCTCTGAATGACTGAATAAGAAGCGAGGCACTAGGATGACGACGATAAGTAGGG	ΔBDLF3.5
ΔBDLF3.5-Rev	TCTCCAAATTCTCTCTCCCCAATAGTGCCTCGCTTCTTATTCAGTCATTCAGAGCATCCGGGGGCAACCAATTAACCAATTCTGATTAG	ΔBDLF3.5
ΔBGLF3-Fwd	TCGGTCAGACGGCCGCGCTGCGAGGCATACAGCATGTTCATAGCTGACTAGGCCGATATGCCCGAAGGATGACGACGATAAGTAGGG	ΔBGLF3
ΔBGLF3-Rev	TACCTGCGGGCGAGCATCGGGTCATCGGGCATATCGGCCTAGTCAGCTATGAACATGCTGTATGCCAACCAATTAACCAATTCTGATTAG	ΔBGLF3
ΔBTRF1-Fwd	AAGACAGCCCCCATCCACTGCCGTGATGCTCAAGTGTAAGTAGCCCTGAGCCCGCTTCATTCACGGGGCTAGGGATAACAGGGTAATCGATTT	ΔBTRF1
ΔBTRF1-Rev	GTCCCGATGGCAGGTGCACGGCCCCGTGAATGAAGCGGGCTCAGGGCTACTTACACTTGAGCATCACGGGCCAGTGTTACAACCAATTAACC	ΔBTRF1
Δ*Ori*Lyt-Fwd	CACTCCTATGCATTTCCTGCCCTCCCACTTTTACCCCAGTTCTATACATTTTCTCAGCACTAGGGATAACAGGGTAATCGATTT	Δ*Ori*Lyt
Δ*Ori*Lyt-Rev	ATGACCCTGATTCATATAAAGTGCTGAGAAAATGTATAGAACTGGGGTAAAAGTGGGAGGGCCAGTGTTACAACCAATTAACC	Δ*Ori*Lyt
Cam-Ff-Kan-Fwd	CGGGCGTATTTTTTGAGTTATCGAGATTTTCAGGAGCTAAGGAAGCTAAAATGAGCCATATTCAACGGGAAAC	Swaps *Chloramphenicol* with *Kanamycin* in F-factor
Cam-Ff-Kan-Rev	CAGGCGTAGCAACCAGGCGTTTAAGGGCACCAATAACTGCCTTAAAAAAATTAGAAAAACTCATCGAGCATC	Swaps *Chloramphenicol* with *Kanamycin* in F-factor

**Table 2:** Primers used for confirmation/sequencing of EBV mutant BACmids.

Primer Name	Sequence	Purpose
ΔBALF2chk-Fwd	CCTTGAAGGCCTCGGTTATT	ΔBALF2/HA-BcRF1
ΔBALF2chk-Rev	GTACAACGACCACTACGACTAC	ΔBALF2/HA-BcRF1
HA-BcRF1chk-Fwd	CACACTCAGGACGGTGTTAAT	ΔBALF2/HA-BcRF1
HA-BcRF1chk-Rev	GAGACAAGTAGCGGATGATAAGG	ΔBALF2/HA-BcRF1
ΔBDLF3.5chk-Fwd	CTGTGTGTCTTGTCGGATCTC	ΔBDLF3.5
ΔBDLF3.5chk-Rev	CCAGAAAGAGGTTGGTGGTT	ΔBDLF3.5
ΔBGLF3chk-Fwd	GTTTGGGAGTGGGCCAATA	ΔBGLF3
ΔBGLF3chk-Rev	GGACACGCTGGTCTTGAAT	ΔBGLF3
ΔBTRF1-Fwd	CGAGGGCCTGTTTGATTTCT	ΔBTRF1
ΔBTRF1-Rev	CATAAGTTGCGAGGAGGCTTTA	ΔBTRF1
Δ*Ori*Lyt-Fwd	AGGTTTAGCTATTCCACCAACA	Δ*Ori*Lyt
Δ*Ori*Lyt-Rev	CGGGTCATTGGCATGTTATTC	Δ*Ori*Lyt

### Derivation of wild-type and mutant EBV-positive 293 cell lines

EBV-positive 293 WT, ΔBALF2/HA-BcRF1, ΔBcRF1, ΔBDLF3.5, ΔBDLF4, ΔBFRF2, ΔBGLF3, ΔBTRF1, ΔBVLF1, and Δ*Ori*Lyt were derived using the BM2710 *E*. *coli*, which can mediate the transfer of intact recombinant DNA into mammalian cells due to expression of the *invasin* gene from *Yersinia pseudotuberculosis* and the *listeriolysin O* gene from *Listeria monocytogenes* [68]. Briefly, BACmids were electroporated using a 0.1 cm gap cuvette (1.5 kV, 200 Ohms, 25 μF) into BM2710 *E*. *coli* and selected with Kanamycin. BM2710 *E*. *coli* containing the respective BACmid were used to infect EBV-negative 293 cells by co-incubation for 2 hours (approximately 25 bacteria per cell). Cell lines were derived by single-cell cloning and, when possible, screened for ability to complete the lytic cascade by immunoblotting for viral late protein VCAp18 after the mutated gene was supplied *in trans*. EBV-positive 293 cells were selected and maintained with 200 μg/ml of Hygromycin B. Viral DNA synthesis was blocked with 100 μg/ml of acyclovir where indicated.

### Plasmids

pcDNA3-Rta, and pSG5-Zta (or pSVNaeZ) have been described previously [69,70]. pGK-Renilla, pMSCV-F-HA-BDLF4, pMSCV-F-HA-BGLF3, and pMSCV-F-HA-BTRF1 have been previously described [71]. pcDNA3-HA(2x)-BVLF1 was constructed by gateway cloning using pDONR223-BVLF1 and pN-2xHA [69]. pcDNA3-HA-BcRF1 was cloned by amplifying *BcRF1* from the EBV genome using primers 5’-CGCGGGTACCGCCACCATGTATCCATATGACGTTCCAGATTACGCTACACAAGGTAAGAGGGAGATG-3’ and 5’-CGCGGAATTCTTACACTTGAGCATCACGGC-3’ and cloning into EcoRI/Acc65I sites of the pcDNA3-HA-SUMO1 vector (Addgene plasmid #21154). pcDNA3-HA-BFRF2 was cloned by amplifying *BFRF2* from the EBV genome using primers 5’-CGCGTGTACAGCCACCATGTATCCATATGACGTTCCAGATTACGCTGCGTTATTCTTGGCGCGCCAC-3’ and 5’-CGCGGAATTCTTAGGAAGCAGGGGACTGTCTGGAAAATC-3’ and cloning into EcoRI/Acc65I sites of the pcDNA3-HA-SUMO1 vector (Addgene plasmid #21154). pSG5-HA-BDLF3.5 was cloned by amplifying *BDLF3*.*5* from the EBV genome using primers 5’- CGCGGAATTCATGTATCCATATGACGTTCCAGATTACGCTTCTGCCCCCGGATGCTCTGAAAG-3’ and 5’-CGCGGGATCCTCAATCGGCCTTGGTCTGAC-3’ and cloning into EcoRI/BamHI sites of the pSG5 vector (Invitrogen). pSG5-BGLF3 was cloned by amplifying *BGLF3* from the EBV genome using primers 5’-CGCGGAATTCATGTTCAACGCGGTCAAGGCCG-3’ and 5’- CGCGGGATCCCTACTCATCTTCATAAGTCAC-3’ and cloning into EcoRI/BamHI sites of the pSG5 vector. pSG5-HA-BALF2 was cloned by amplifying the *BALF2* from EBV genome using primers 5’-CGCGGAATTCATGTATCCATATGACGTTCCAGATTACGCTCAGGGTGCACAGACTAGCGAGG-3’ and 5’-CGCGGGATCCCTAGACCTCGAGTCCGGGGAG-3’ and cloning into EcoRI/BamHI sites of the pSG5 vector. pGL2-TATT was cloned by annealing the oligos 5’- CCGGGTAT**T**AAACCGGGTGGCAGCTCCTGGCAGTCATTCAC-3’ and 5’- TCGAGTGAATGACTGCCAGGAGCTGCCACCCGGTTT**A**ATAC-3’ and cloning into XmaI/XhoI sites of the pGL2-Basic vector. pGL2-TATA is identical to pGL2-TATT except with a point mutation changing the T at the fourth position in the TAT**T** sequence to an A and was constructed by annealing the oligos 5’-CCGGGTAT**A**AAACCGGGTGGCAGCTCCTGGCAGTCATTCAC-3’ and 5’-TCGAGTGAATGACTGCCAGGAGCTGCCACCCGGTTT**T**ATAC-3’ and cloning into XmaI/XhoI sites of the pGL2-Basic vector. pGL2-TATT-*Ori*Lyt is similar to pGL2-TATT with addition of EBV minimal *Ori*Lyt downstream of the Luciferase gene and was constructed first by annealing the oligos 5’-TAGTCGAGCTCAGGAGAATTCATTGATGCATG-3’ and 5’-TCGACATGCATCAATGAATTCTCCTGAGCTCGACTA-3’ into SalI/PshAI sites of pGL2-TATT to create a multiple cloning site followed by cloning the minimal *Ori*Lyt DNA fragment cut with NsiI/SacI from the B95.8 BamHI-H fragment into the resulting plasmid. pTATT-GFP-VCAp18-*Ori*Lyt, which contains EBV full *Ori*Lyt and GFP-VCAp18 under its native promoter was constructed as follows: The *BFRF3* gene was amplified with the primer pair 5'-ATCGGAATTCATGGCACGCCGGCTGC-3' and 5'-ATGCTACTCGAGCTGTTTCTTACGTGCCCCGCGTGG 3’, inserted into the EcoRI/SalI sites of pEGFP-C2 (Clontech) after digestion with EcoRI and XhoI to generate pEGFP-VCAp18. The *BFRF3* promoter was amplified with the primer pair 5’-GAATGCATTAATGTTATTCTTGGCGCGCCACACCT 3’ and 5’-ATCGCAGCTAGCAACGCGTCTGATAGAGACGGCAGC 3’, digested with AseI and NheI, and inserted into the AseI/NheI site of pEGFP-VCAp18 to replace the CMV promoter and generate pBFRF3p-gfpBFRF3. The DNA fragment encoding *BFRF3* promoter and GFP-VCAp18 fusion was isolated from pBFRF3p-gfpBFRF3 with AseI and SspI, blunt-ended using Klenow, and inserted into the Klenow-filled HindII site of p562 [20] to generate pTATT-GFP-VCAp18-*Ori*Lyt, in which the *BFRF3* promoter drives the expression of GFP-VCAp18 fusion towards the replication origin, *Ori*Lyt, for EBV's lytic genome amplification. pTATT-GFP-VCAp18 is a modification of pTATT-GFP-VCAp18-*Ori*Lyt without the full *Ori*Lyt fragment and was constructed by cutting pTATT-GFP-VCAp18-*Ori*Lyt with SfiI/SalI followed by Klenow treatment and unimolecular ligation. In order to create pTAT**A**-GFP-VCAp18, a 222 bp and a 964 bp fragment were PCR amplified from the pTATT-GFP-VCAp18 using the primers (p4222-Blp-side-F: 5’-GCGGCGGTGGGCTGCCAGAG-3’; p4222-TATA-R: 5’-CCGCAAAGTTA**T** **ATA**CAGGAGCTGCCTGACC-3’) and (p4222-TATA-F: 5’-CAGCTCCTG**TAT** **A**TAACTTTGCGGACAGAGGC-3’; p4222-Xho-side-R: 5’-AGCCGGCGTGCCATGAATTC-3’) respectively and the pTAT**A**-GFP-VCAp18 was constructed via Gibson Assembly (New England Biolabs) using the two PCR products and the original pTATT-GFP-VCAp18 vector backbone (cut with BlpI and XhoI). The integrity of the final plasmid was initially confirmed by restriction digestion with multiple enzymes (including MseI, which cleaves the sequence 5’-…T**|**TAA…-3’ distinguishing between TATA and TATT) and subsequently sequencing of the appropriate junctions.

### Immunoblotting

Total cell lysates were harvested in Laemmli Buffer and separated by sodium dodecyl sulfate polyacrylamide gel electrophoresis (SDS-PAGE), and transferred to nitrocellulose membrane. Membranes were blocked in Tris-buffered saline (TBS) containing 5% milk and 0.1% Tween 20 and incubated with appropriate primary antibodies overnight at 4°C. The following primary antibodies were used: anti-EBV EA-D p52/50 (EMD Millipore, MAB8186; 1:3,000), anti-EBV VCA-p18 (Thermo Scientific, PA1-73003; 1:1,000), anti-EBV Zta (Santa Cruz Biotechnology, sc-53904; 1:250), anti-EBV Rta (Thermo Scientific, custom antibody described here [72]; 1:1000), anti-HA (Covance, MMS-101P; 1:1,000), and anti-α-tubulin (Sigma, T6074; 1:1,000). Following treatment with primary antibodies, membranes were washed with TBS containing 0.1% tween and incubated with appropriate secondary antibodies for 1 hour at room temperature. The following secondary antibodies were used: goat anti-mouse poly-HRP (Fisher Scientific), goat anti-rabbit poly-HRP (Fisher Scientific), and donkey anti-goat (Fisher Scientific). Membranes were washed again and visualized on film using ECL chemiluminescent kit (Thermo Scientific) according to manufacturer’s protocol.

### Reporter assays

Cells were transfected in 12-well plates with 0.25 μg of reporter plasmid, 0.25 μg of *trans*-complementing plasmid where indicated, 0.02–0.125 μg of R and Z expression plasmid, and 0.025 μg of a plasmid expressing renilla luciferase (as a control) using Lipofectamine 2000 (Invitrogen) according to manufacturer’s protocol. After 48 hours, cells were lysed in passive lysis buffer (Promega) for 15 minutes at room temperature on a rocking platform and clarified by centrifugation. Firefly and renilla luciferase values were measured using the dual-luciferase reporter assay kit (Promega) on a BD Monolight 3010 luminometer (BD Biosciences).

### Quantification of viral DNA copy number by real-time (RT) PCR

EBV-positive 293 cells were induced in 12-well plates using 125 ng each of R and Z expression plasmids along with 250 ng of the *trans*-complementing plasmid. 48 hours post lytic induction, cells were washed with phosphate-buffered saline (PBS), and genomic DNA was extracted using GeneJET genomic DNA purification kit (Thermo Scientific) according to manufacturer’s protocol. Purified DNA was subjected to qRT-PCR with a 7900HT Fast Real-Time PCR system (Applied Biosciences) using SYBR Green Real-Time PCR Master Mix (Biorad) with primers specific to EBV *Ori*Lyt (5’-TCGCCTTCTTTTATCCTCTTTTTG-3’ and 5’-CCCAACGGGCTAAAATGACA-3’) and GAPDH (5’-CTCCCGCTTCGCTCTCT-3’ and 5’-TTTCTCTCCGCCCGTCTT-3’). All values were normalized to GAPDH using the 2^-ΔΔ*C*^_T_ method described previously [73].

### Single-molecule RNA fluorescence in situ hybridization

RNA fluorescence *in situ* hybridization (FISH) was performed following manufacturer’s protocol (LGC Bioresearch Technologies). The probes employed are single-stranded DNA oligos (20 nucleotides), each labeled with the fluorophore Quasar 670 and were designed using online Stellaris probe designer provided by manufacturer (LGC Bioresearch Technologies). i293 Visible EBV cells [47] were plated on coverslips and grown overnight at 37°C. Replication was induced by addition of 200nM 4-hydroxytamoxifen and LacI-tdTomato binding to the EBV genome induced by IPTG withdrawal. At 36–48 hours post-induction, cells were washed once with PBS and fixed with 3.7% formaldehyde/PBS solution for 10 minutes at room temperature. Next, cells were washed twice with PBS and subsequently permeabilized with 70% ethanol at 4°C, overnight. The cells were then washed with Wash Buffer A (LGC Bioresearch Technologies) at room temperature for 5 minutes before hybridization. To detect viral RNAs, 125 nM of labeled probes in 100 μl of Hybridization Buffer (LGC Bioresearch Technologies) was used for each sample. Hybridization was carried out in humidified chambers maintained at 37°C for 16 hours. The samples were then washed twice with Wash Buffer A at 37°C for 30 minutes, and once with Wash Buffer B (LGC Bioresearch Technologies) at room temperature for 5 minutes. Nuclear staining was performed using Vectashield Antifade mounting medium with DAPI (Vector Laboratory Inc.). The distributions of EBV mRNAs and amplified genomes in the lytic cycle were imaged with a Zeiss Apotome microscope.

### RNA isolation, reverse transcription and quantification by real-time (RT) PCR

EBV-positive 293 cells were induced in 12-well plates using 125 ng each of R and Z expression plasmids along with 250 ng of the *trans*-complementing plasmid. 48 hours post lytic induction, cells were washed with phosphate-buffered saline (PBS), and RNA was extracted using GeneJET RNA purification kit (Thermo Scientific) according to manufacturer’s protocol with the following modification: after lysis and before loading on column, lysates was passed through a QIAshredder cell and tissue homogenizer (Qiagen). The eluted RNA was then treated with DNase (1 unit/μg DNA), DNase was deactivated by incubation at 65°C and the treated RNA (~ 1 μg) was reverse transcribed using the ImProm-II Reverse Transcription System (Promega). Purified cDNA was subjected to qRT-PCR with a 7900HT Fast Real-Time PCR system (Applied Biosciences) using SYBR Green Real-Time PCR Master Mix (Biorad). The primers used to detect various EBV transcripts and β-Actin are listed in S1 Table.

## Supporting Information

S1 Fig Six EBV βγ genes are expressed with early kinetics. Bar plots showing mRNA levels of six βγ genes (*BcRF1*, *BDLF3*.*5*, *BDLF4*, *BGLF3*, *BFRF2*, and *BVLF1*) relative to β-Actin in 293 EBV ΔBALF2/HA-BcRF1 cells that were uninduced (U), induced (I) by transfection with R and Z expression plasmids, or induced and *trans*-complemented by transfection with plasmid expressing R, Z, and BALF2 (I + *t*) at 48 hours post induction. Transcripts for six βγ genes are detected in the induced condition (I, middle bar) and were not significantly increased by *trans*-complementation with BALF2 (I + t).
(TIF)

S1 Table Primers used for detection of cDNAs corresponding to 6 essential βγ transcripts and the β-Actin reference. (PDF)
