# Supplementary figures and images for: An Epstein-Barr Virus-Encoded Protein Complex Requires an Origin of Lytic Replication In Cis to Mediate Late Gene Transcription
Source: PLoS Pathog. 2016 Jun 27;12(6):e1005718. doi: 10.1371/journal.ppat.1005718 (PMC4922670; doi:10.1371/journal.ppat.1005718)

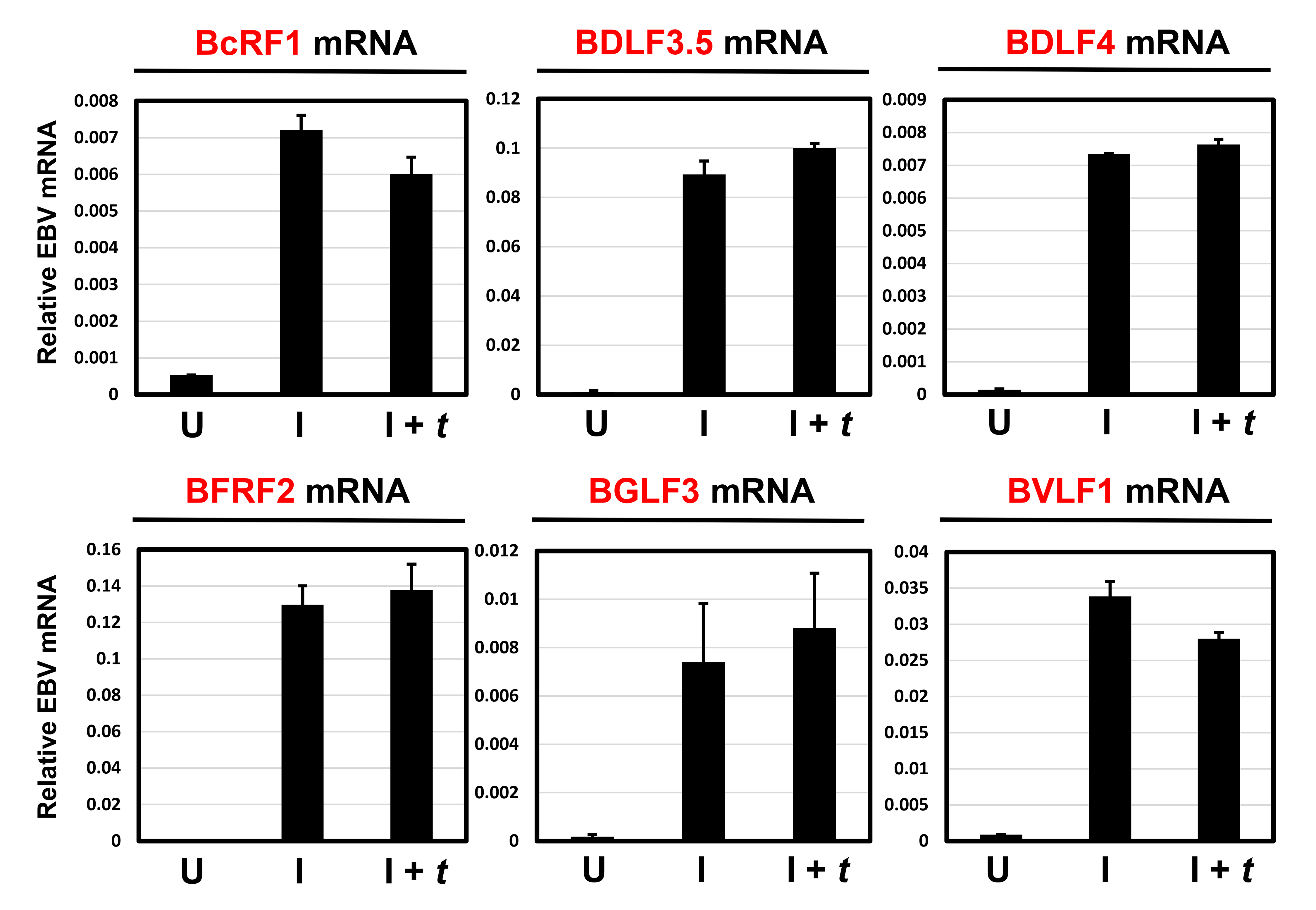

Supplement: S1 Fig — Bar plots showing mRNA levels of six βγ genes (BcRF1, BDLF3.5, BDLF4, BGLF3, BFRF2, and BVLF1) relative to β-Actin in 293 EBV ΔBALF2/HA-BcRF1 cells that were uninduced (U), induced (I) by transfection with R and Z expression plasmids, or induced and trans-complemented by transfection with plasmid expressing R, Z, and BALF2 (I + t) at 48 hours post induction. Transcripts for six βγ genes are detected in the induced condition (I, middle bar) and were not significantly increased by trans-complementation with BALF2 (I + t). (TIF) [file ppat.1005718.s001.tif]
