# Supplementary material for: An Epstein-Barr Virus-Encoded Protein Complex Requires an Origin of Lytic Replication In Cis to Mediate Late Gene Transcription
Source: PLoS Pathog. 2016 Jun 27;12(6):e1005718. doi: 10.1371/journal.ppat.1005718 (PMC4922670; doi:10.1371/journal.ppat.1005718)
Supplement: S1 Table — (PDF) [file ppat.1005718.s002.pdf]

Primer Name	Sequence	Target cDNA
BcRF1-cDNA-Fwd	GCGTTCACCGTCAGTCTATT	BcRF1
BcRF1-cDNA-Rev	GGCCAATAGGAAGCTCAGATAG	BcRF1
BDLF3.5-cDNA-Fwd	AGAGACCTCCTGCTCAAAGA	BDLF3.5
BDLF3.5-cDNA-Rev	GGATAAGAAGCGAGGCACTATT	BDLF3.5
BDLF4-cDNA-Fwd	CTTGTTCAGGCTCCCACTT	BDLF4
BDLF4-cDNA-Rev	GCCCTTACAGGCAACTACAT	BDLF4
BGLF3-cDNA-Fwd	GGCTCGAGAAGTCATAGTCATC	BGLF3
BGLF3-cDNA-Rev	TTCCTGCTCGTGTCCAATATC	BGLF3
BFRF2-cDNA-Fwd	ATCATGCCTCGGCTCTATTG	BFRF2
BFRF2-cDNA-Rev	CTCCTCAGGCACGAGTTTAC	BFRF2
BVLF1-cDNA-Fwd	GACACTGCCAAGGTTGAAGA	BVLF1
BVLF1-cDNA-Rev	CCGTCTGTGCATGTGTGT	BVLF1
β-Actin-cDNA-Fwd	GCCGGGACCTGACTGACTAC	β-Actin
β-Actin-cDNA-Rev	TTCTCCTTAATGTCACGCACGAT	β-Actin
